# The Nature of Functional Features of Different Classes of G-Protein-Coupled Receptors

**DOI:** 10.3390/biology11121839

**Published:** 2022-12-16

**Authors:** Ke An, Xiaohong Zhu, Chen Bai

**Affiliations:** 1 Warshel Institute for Computational Biology, School of Life and Health Sciences, School of Medicine, The Chinese University of Hong Kong, Shenzhen, Shenzhen 518172, China; 2 School of Chemistry and Materials Science, University of Science and Technology of China, Hefei 230026, China; 3 Chenzhu (MoMeD) Biotechnology Co., Ltd., Hangzhou 310005, China

**Keywords:** GPCR, energy landscape, structure–function relationship

## Abstract

**Simple Summary:**

The sequence–structure–function paradigm, which emphasizes the relationship between the 3D structure and functions of a protein, is a core concept in biology. The relationship further determines the functional specificity of proteins. By studying G-protein-coupled receptors (GPCRs), we investigated how the 3D structures of proteins are related to the activation mechanisms and functions. The activation of GPCRs involves many events, such as conformational changes, agonist binding, G-protein binding, nucleotide binding/release, etc. By exploring the coupled free-energy landscape of these events, we offer a possible explanation for the functional differences between the three families of GPCRs and how they are related to their signal responding specificities.

**Abstract:**

G-protein-coupled receptors (GPCRs) are a critical family in the human proteome and are involved in various physiological processes. They are also the most important drug target, with approximately 30% of approved drugs acting on such receptors. The members of the family are divided into six classes based on their structural and functional characteristics. Understanding their structural–functional relationships will benefit us in future drug development. In this article, we investigate the features of protein function, structure, and energy that describe the dynamics of the GPCR activation process between different families. GPCRs straddle the cell membrane and transduce signals from outside the membrane into the cell. During the process, the conformational change in GPCRs that is activated by the binding of signal molecules is essential. During the binding process, different types of signal molecules result in different signal transfer efficiencies. Therefore, the GPCR classes show a variety of structures and activation processes. Based on the experimental crystal structures, we modeled the activation process of the *β*2 adrenergic receptor (*β*2AR), glucagon receptor (GCGR), and metabotropic glutamate receptor 2 (mGluR2), which represent class A, B, and C GPCRs, respectively. We calculated their activation free-energy landscapes and analyzed the structure–energy–function relationship. The results show a consistent picture of the activation mechanisms between different types of GPCRs. This could also provide us a way to understand other signal transduction proteins.

## 1. Introduction

G-protein-coupled receptors (GPCRs) represent the largest membrane receptor family in the human body and consist of about 800 members encoded by the human genome [1,2]. They recognize various types of extracellular signaling molecules, such as odors, hormones, neurotransmitters, and chemokines. GPCRs transduce their signals across the membrane to trigger intracellular responses [2,3]. GPCRs have crucial roles in various physiological and pathological processes, including proliferation, differentiation, chemotaxis, and communication [4]. Because of this, they have been recognized as promising drug targets, and many drugs have been approved by the FDA (527 drugs) [5] or are in clinical trials (~60 drug candidates) [6].

All GPCRs share a transmembrane domain (TMD) with a common structural architecture and present similar conformational changes during activation. The TMD consists of a bundle of seven α-helices that are embedded in the cell membrane and linked by intracellular and extracellular loops [7,8]. The signaling ligands (agonists) bind to the extracellular binding site and favor structural changes that allow G proteins or other signaling proteins to bind to the intracellular surface [9]. One hallmark of GPCR activation is the outward movement of the cytoplasmic end of transmembrane domain 6 (TM6), which opens an intracellular cavity to accommodate the Gα subunit, leading to nucleotide exchange and the activation of the G protein [10]. Beyond the similarity, GPCR structures also have diversity between different classes. GPCRs can be grouped into six classes (classes A–F) based on sequence homology and functional similarity [1,2]. They have their own structural characteristics, such as the additional eighth helix at the C terminal of class A, the larger extracellular domain of class B, and the multiple structural domains of class C [1,2]. These structural differences also affect the dynamic process of GPCR activation.

The activation of GPCRs is considered a conformational transfer process from the inactive to the fully activated state [3,9]. Over the last two decades, structural biologists have provided a large number of GPCR-G protein complex and GPCR-agonist complex structures, providing a wealth of material for understanding the mechanisms [11,12,13]. The ever-growing evidence demonstrates that multiple conformational states (intermediates) exist in the activation of GPCRs to concatenate the inactive and fully activated states rather than the initial understanding as simple ON/OFF molecular switches [14]. Therefore, it is efficient and necessary to determine the intermediate states and use energy landscapes to conceptualize the link between the structure and function of GPCRs [15]. A series of schematic and conceptual dynamics models of GPCR activation have been proposed [3,9,15]. However, it is still difficult to quantitatively characterize the energy landscapes of GPCR activation due to the large size of the system and the complexity of the conformational change process.

Physically rational simplifications of models have produced a dramatic benefit in modeling large-scale biophysical complex systems [16]. The GPCR proteins usually consist of thousands of amino acids, limiting the application of all-atom (AA) models in modeling and simulating GPCR systems. The idea of such coarse-graining has become a common, well-accepted, and powerful strategy [16]. Here, we investigated the activation process of GPCRs using a coarse-grained (CG) model [17,18,19,20], which has been proven to be very effective in investigating many biophysical systems such as ATPase [21,22], SARS-CoV-2 spike [23,24], and GPCRs [25,26,27]. The CG strategies for a description of the functional properties focused on improving the electrostatic features of the model [17] and were refined for the treatment of membrane proteins [18]. Moreover, the CG model has been shown to be applicable in mechanistic studies related to conformational changes in large-scale protein machines [20].

From the energy landscape perspective, GPCR dynamics can be described as one-dimensional or two-dimensional energy diagram projections of several conformational states along the principal activation coordinates. In this study, we modeled the activation process of the β2 adrenergic receptor (*β*2AR, class A), glucagon receptor (GCGR, class B), and metabotropic glutamate receptor 2 (mGluR2, class C). We generated the structural models of the end conformations and simulated the converting process to obtain the intermediate structures using the target molecular dynamics approach. Considering the conformational change trajectories of GPCRs as the principal activation coordinates, we obtained their activation energy landscapes and energy barriers. By calculating the energy barriers in activated conditions, we discuss the functional, structural, and energy relations of these GPCRs.

## 2. Materials and Methods

### 2.1. Modeling the Structures

The structures modeled in this study all followed the workflow we reported previously [20]. First, the structures of the inactive, intermediate, and active states were extracted from corresponding experimental structures, which were as follows: *β*2AR-inactive (PDB ID: 6EG8), *β*2AR-intermediate (PDB ID: 6E67), *β*2AR-active (PDB ID: 3SN6), GCGR-partially active (PDB ID: 5YQZ), GCGR-fully active (PDB ID: 6WPW), mGluR2-inactive (PDB ID: 7EPA), and mGluR2-fully active (PDB ID: 7E9G). The missing parts were repaired by Modeller software [28]. All structures of different conformational states of a protein were trimmed to the same length to maintain the consistency of residues. After that, a targeted molecular simulation [29] was conducted to construct the conformational pathways between different endpoint structures and sample a series of intermediate conformations representing the transition process. After obtaining the intermediate conformations, we added membrane particles at the TMD of each structure and performed extensive molecular dynamics relaxation using Molaris-XG [16,30] until the energy converged.

### 2.2. Calculating the Folding Free Energy of Protein

The all-atom structures were converted into a CG representation and subjected to extensive relaxation. Our CG model, adapted from previous studies [17,18,19], focused on the precise treatment of the electrostatic charges and was sensitive to the charge distribution of the ionized groups in the protein. Hence, before the energy evaluation, a Monte Carlo proton transfer (MCPT) method [18] was used to determine the charge states of the residues in each structure. During the MCPT, protons “jumped” between ionizable residues, and a standard Metropolis criterion was utilized to calculate the acceptance probability. The total CG folding free energy ($\Delta G_{fold}$) was calculated according to the following formula:

(1) $$\begin{array}{c} \Delta G_{fold}=\Delta G_{main}+\Delta G_{side}+\Delta G_{main-side}\\ =c_{1}\Delta G_{side}^{vdw}+c_{2}\Delta G_{solv}^{CG}+c_{3}\Delta G_{HB}^{CG}+\Delta G_{side}^{elec}+\Delta G_{side}^{polar}+\Delta G_{side}^{hyd}+\Delta G_{main-side}^{elec}\\ \;\;\;\;\;\;\;+\Delta G_{main-side}^{vdw} \end{array}$$

In this formula, the CG folding free energy ($\Delta G_{fold}$) consists of three parts: the main-chain free energy ($\Delta G_{main}$), the side-chain free energy ($\Delta G_{side}$), and the free energy of the main–side interactions ($\Delta G_{main-side}$). These three parts can also be divided into eight terms: the side-chain van der Waals energy ($\Delta G_{side}^{vdw}$), main-chain solvation energy ($\Delta G_{solv}^{CG}$), main-chain hydrogen bond energy ($\Delta G_{HB}^{CG}$), side-chain electrostatic energy ($\Delta G_{side}^{elec}$), side-chain polar energy ($\Delta G_{side}^{polar}$), side-chain hydrophobic energy ($\Delta G_{sode}^{hyd}$), main-chain/side-chain electrostatic energy ($\Delta G_{main-side}^{elec}$), and main-chain/side-chain van der Waals energy ($\Delta_{main-side}^{vdw}$). The scaling coefficients c1, c2, and c3 were set as 0.10, 0.25, and 0.15, respectively.

Each structure used for the energy calculation performed an extensive molecular dynamics relaxation. Then, the last ten conformations of the relaxation trajectory were used to calculate their folding energies. We used the energy of the last conformation to draw the figure and conduct the analysis. In addition, the standard deviations of the ten energies were used as the error bar. All relative calculations were performed using the Molaris-XG package [16,30].

### 2.3. Ligand Docking and Calculating the Binding Free Energy

According the experimental structure (PDB ID: 7DHR for *β*2AR [31] and PDB ID: 7EPB for mGluR2 [32]), we first defined the binding pockets of the two ligands (adrenaline and glutamate). Then, the docking was performed using the LigPrep and Glide tools in Schrödinger.

To calculate the binding free energy, some parameters of the two ligands were added into the Molaris-XG force field, including atom names, atom types, atom charges, and bond descriptions. The most important term, atom charges, was calculated using Gaussian software.

The binding free energy values were calculated by the PDLD/s-LRA/*β* method [33,34,35]. Scaled protein dipole Langevin dipole (PDLD/S) is a method to calculate the electrostatic energies of a system at a semi-microscopic level to maintain the benefits of microscopic and macroscopic models. In the PDLD/S model, the solvent molecules were represented by a grid of Langevin dipoles (LD) to consider the average polarization of the solvent. The averaging in PDLD/S was performed using linear response approximation (LRA). The non-electric binding contribution (a scaled vdW term) was approximated using the linear interaction (LIE) method. Thus, the overall method was called PDLD/S-LRA/*β*, where *β* is the scale of vdW.

## 3. Results

### 3.1. Agonist Binding Reduces the Activation Energy Barrier of β2AR Adrenergic Receptor

**Figure 1:**
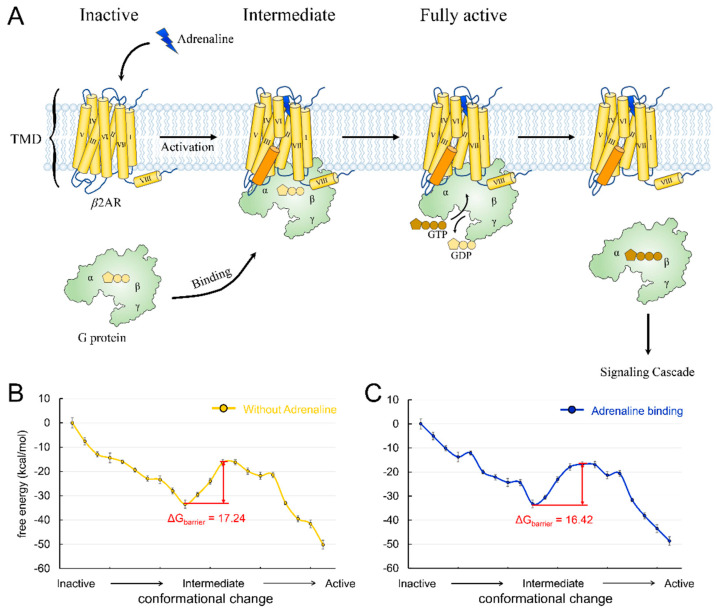
(**A**) Schematic description of *β*2AR structure and activation pathway. *β*2AR is shown in yellow, and its TM6 is in orange. The adrenaline is shown as a flash in blue. The G protein is shown in light green. (**B**) The energy landscape of the conformational change of *β*2AR without adrenaline binding. (**C**) The energy landscape of the conformational change of *β*2AR with adrenaline binding to each conformation.

Class A GPCRs, so called “rhodopsin-like” receptors, are the largest GPCR class. The human genome encodes 719 class A members [2]. Typically, class A GPCRs have a TMD that forms a ligand-binding pocket, a short disordered N-terminal region, and additional eighth helices with palmitoylated cysteines at the C terminals [1,2]. Roughly half of class A GPCRs are sensory receptors involved in smell (pheromone receptors) or vision (rhodopsins), and about 350 non-sensory receptors of class A are activated by diffusible ligands such as hormones or neurotransmitters. The first crystal structure of GPCR extracted from exogenously expressed host cells was the complex structure of *β*2AR with its antagonist [36]. *β*2AR is a non-sensory receptor activated by adrenaline that regulates many physiological processes, including cardiac function, airway tone, and metabolic functions [37]. The receptor is frequently used as a representative for the study of GPCR activation mechanisms. The activation of *β*2AR starts with adrenaline binding to the ligand-binding pocket, followed by the hallmark movement of TM6 to form an intracellular cavity (Figure 1A). The Gs protein carrying guanosine diphosphate (GDP) binds to the cavity. Then, the α domain of Gs opens. Thus, GDP is released, and GTP is bound. Finally, Gs is released, and signal transduction is complete (Figure 1A). Previously, we have investigated the coupling between the *β*2AR conformational change and nucleotide release [27]. However, the effect of agonist binding on *β*2AR activation is not well understood.

We constructed the conformational change trajectory of *β*2AR from an inactive state to an intermediate state and then to an active state and calculated the energy barriers with or without adrenaline binding. The inactive, intermediate, and active state conformations came from their crystal structures (PDB ID: 6EG8, 6E67, and 3SN6). A series of conformations between each state was generated by targeted molecular dynamics, and ten structures at equal intervals were picked for each transition. The energy landscapes showed that the energy barrier of the conformational change is 17.24 kcal/mol (Figure 1B) and that adrenaline binding reduces the energy barrier to 16.42 kcal/mol (Figure 1C). The barrier difference mainly comes from the interaction between adrenaline and the threonine located on extracellular loop 2 (T188). We mutated the threonine to alanine and calculated the change in the energy barrier. The result showed that the mutation (T188A) reduced the energy barrier to 14.10 kcal/mol. In addition, we also identified a key residue (D113) playing an essential role in agonist binding to *β*2AR, which was consistent with previous reports [31,38,39,40]. We also introduced mutation D113A into the receptor and found that the energy barrier of *β*2AR activation increased to 19.15 kcal/mol. The results suggested a facilitative effect of agonist binding on receptor activation, consistent with the classical *β*2AR activation model [3,15]. *β*2AR exhibits a high signal transmission efficiency, which is demonstrated by the faster G-protein association rate, faster nucleotide release rate, and higher guanine nucleotide exchange factor activity [10]. This high efficiency meets the requirements of the human body for a rapid response to adrenaline. The facilitative effects of agonists on *β*2AR activation can partly explain the high signal transmission efficiency of the receptor. A similar mechanism may also exist in other class A GPCRs, such as sensory receptors, which also require a rapid response to sensory signals.

### 3.2. G protein Approaching Plays an Essential Role in Glucagon Receptor Activation

**Figure 2:**
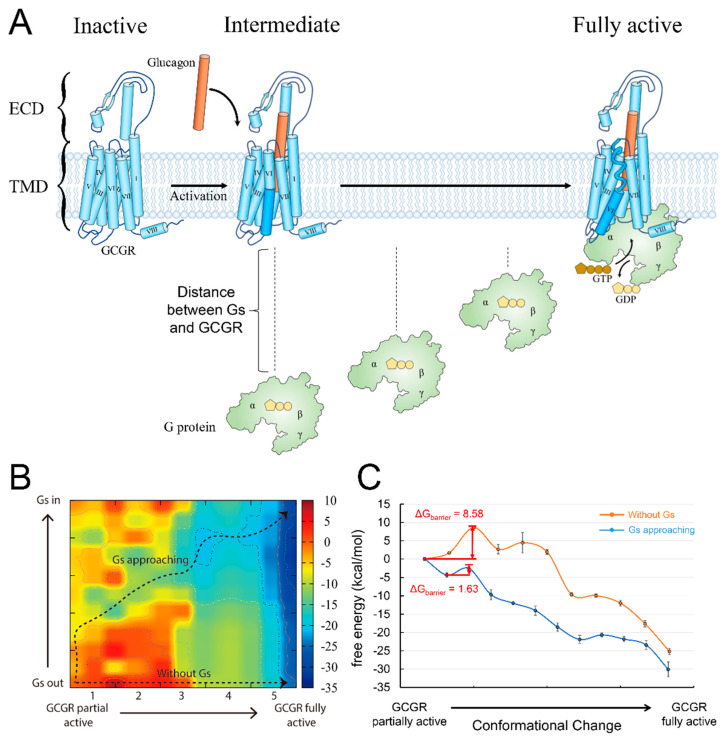
(**A**) Schematic description of GCGR structure and activation pathway. GCGR is shown in light blue, and its TM6 is in dark blue. The glucagon peptide is shown as a cylinder in orange. The G protein is shown in light green. (**B**) The two–dimensional energy map coupling the Gs distance and the conformational change in GCGR from the partially active state to the fully active state. The X axis is the conformational change coordinate, while the Y axis denotes the Gs distance from the receptor. The two black dotted lines with arrows represent the energetic pathways. (**C**) The energy landscape of the corresponding pathway in the two-dimensional energy map.

GCGR is a prototypical class B GPCR that is activated by the peptide hormone glucagon and then interacts with the adenylyl cyclase stimulatory G protein, Gs, leading to increased cyclic adenosine monophosphate (cAMP) production [41]. Moreover, GCGR is constitutively ubiquitinated at the cell surface [42]. When GCGR is stimulated by glucagon, it not only promotes the endocytic trafficking of GCGR but also induces rapid deubiquitination. This process has a significant effect on the trafficking and signaling of GCGR. GCGR contains a large extracellular domain (ECD) containing a binding pocket for its agonist, the glucagon peptide (Figure 2A). In the inactivated state, the C-terminal part of the glucagon peptide interacts with the ECD, and the N-terminal peptide inserts into the TMD (Figure 2A) [14]. The binding actuates the deubiquitylation of GCGR and drives GCGR’s conformational changes, including the landmark outward movement of the cytoplasmic end of transmembrane domain 6 (TM6) and the conformational rearrangement of the other parts [10,42]. In the fully active state, GCGR’s TM6 moves outward by ~18 Å, which is larger than the 14 Å movement of *β*2AR [10]. People have resolved the structure of the partially active state conformation between the inactive and fully activated states of GCGR [10]. In contrast to class A GPCR, with only agonist binding, TM6 cannot move outward enough to form the binding pocket for the Gs protein (Figure 2A). The complete movement of TM6 requires a sharp kink (105.5°) formation in the middle of TM6, which creates an energy barrier for GCGR activation.

G protein engagement is proposed to help overcome the energy barrier [10]. To investigate this, we calculated the energy barrier for the conformational change in GCGR from the partially active state to the fully active state with or without Gs approaching. The conformation of GCGR in the partially active state came from the crystal structure of the receptor–partially active agonist complex (PDB ID: 5YQZ) [43]. In addition, the fully active GCGR conformation was obtained from the Cryo-EM structure of a GCGR-Gs complex activated by a full agonist (PDB ID: 6WPW) [10]. When the Gs protein was far away from the receptor, the energy barrier of the conformational change of GCGR was 8.58 kcal/mol (Figure 2C), which was consistent with the kink formation of TM6. To model the effect of the G protein’s approach on the energy barrier, we pulled Gs away from its binding site to different distances from GCGR, thus constructing a two-dimensional (2D) energy map by coupling the Gs distance and the GCGR conformational change (Figure 2B). As a result, we found a pathway from the partially active GCGR with the non-bound Gs (bottom left of the 2D map, Gs protein is far away from GCGR) to the fully active GCGR-Gs complex (top right of the 2D map, Gs has formed a complex with GCGR). The pathway describes the process through which Gs gradually approaches GCGR and eventually binds to the receptor. It has a lower energy barrier, 1.63 kcal/mol, than the barrier of GCGR activation without the Gs protein approaching (Figure 2C). The result indicates that the G protein approach indeed promotes GCGR activation.

The current results are compatible with the function feature of GCGR. GCGR plays an important role in regulating blood glucose levels and thus represents a therapeutic potential for obesity and type 2 diabetes therapies [44,45]. The full activation of GCGR requires not only agonist binding but also G-protein induction, thus exhibiting less activation efficiency, in contrast to *β*2AR, which only needs to bind to an agonist molecule before reaching the activation state [10]. After GCGR reaches the fully active state (the dark blue area in the right part of Figure 2B), its conformation can be maintained at a lower energy level, even in a state without G protein. The result was consistent with the observation that GCGR can maintain its activity for a prolonged time after Gs protein dissociation [10]. Therefore, the signal curve of GCGR is flatter and without a significant energy barrier. These features enable animals’ bodies to withstand the regulation of blood glucose by glucagon and avoid steep increases or decreases in blood glucose levels.

### 3.3. Two Agonists Are Required for the Activation of Metabotropic Glutamate Receptor 2

**Figure 3:**
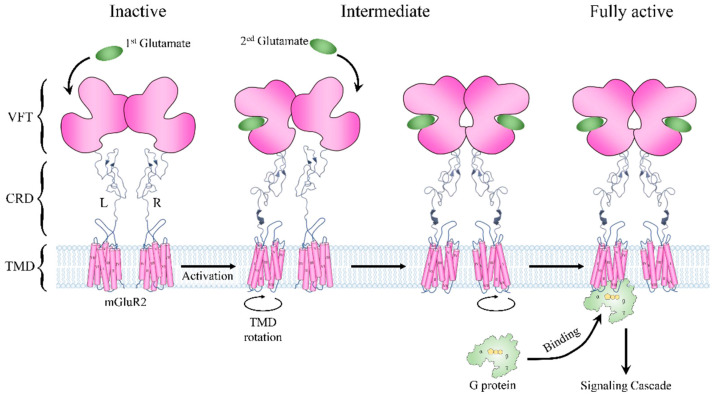
Schematic description of mGluR2 structure and activation pathway. The receptor is shown in pink. L and R represent its two subunits. The glutamate molecule is represented by an oval in green. The G protein is shown in light green.

The metabotropic glutamate receptors (mGluRs) are archetypal class C GPCRs containing eight members (mGluR1-8) [46]. Like other GPCRs, mGluRs contain TMDs consisting of seven helices. Their distinctive structural features are the large ECD, consisting of a Venus fly trap (VFT) part and a cysteine-rich domain (CRD), and an obligated constitutive dimer for receptor activation (Figure 3) [47]. A VFT domain consists of two lobes that sit one atop the other, and agonists bind in a cleft between them (Figure 3) [46]. In the absence of glutamate, the mGluR2 dimer remains in an inactivate state that involves an asymmetric interface. The VFD domains of the receptor adopt an open conformation. Once the glutamate binds to the VFT domain, it can induce a substantial compaction of the receptor dimer, and the VFT and the CRD domain move close to their counterparts in the other subunit. Then, signals are delivered to the TMDs, leading to the rotation of the two TMDs and generating a dimer interface along with helix VI (Figure 3) [48,49]. Two glutamate signal molecules are required for the full activity of an mGluR dimer. Moreover, to facilitate G-protein coupling, the TMDs undergo a further movement to introduce an asymmetric dimer interface. However, only one TMD is responsible for G-protein binding, which suggests an asymmetric signal transduction mode in mGluRs (Figure 3) [50,51]. The structural basis of the mode is that the simultaneous activation of both TMDs in the dimer and the binding of two G proteins would result in a severe clash [52]. However, currently, it is not clear which TMD would bind to the G protein and transmit the signal. Considering that mGluRs are widely expressed in neurons and astrocytes and are responsible for neural signaling [46], the asymmetric activation mode may originate from the need to verify input signals, thus avoiding the abnormal activation of neuronal cells.

**Figure 4:**
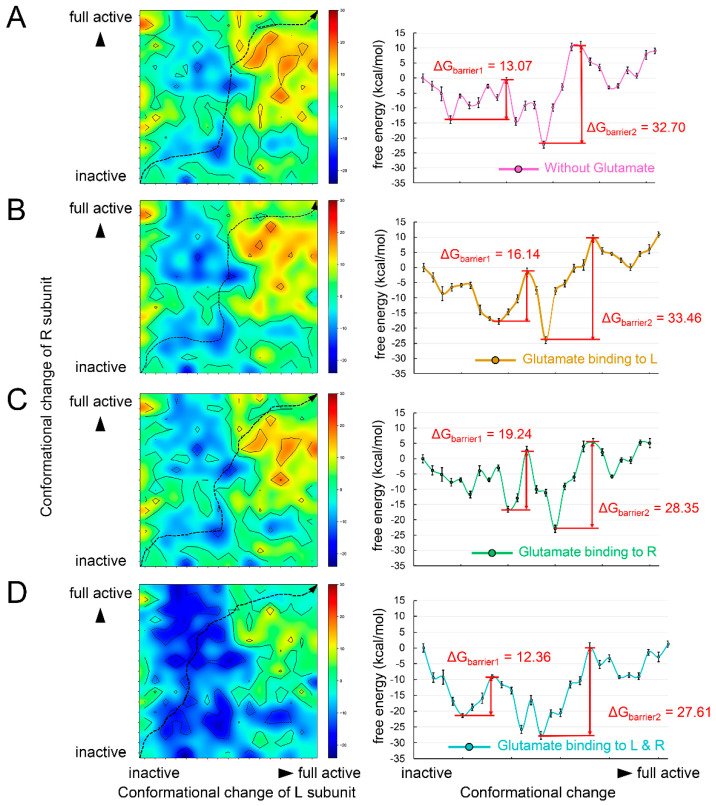
The energy maps of the two–dimensional conformational change system and the energy landscapes of its least energy pathway under the conditions (**A**) without any glutamate binding, (**B**) with glutamate binding to the L subunit, (**C**) with glutamate binding to the R subunit, and (**D**) with 2 glutamates binding to the L and R subunits.

In order to validate the energy mechanism of the asymmetric activation of mGluRs, we constructed structural dynamics models from an inactive state to a fully active state using mGluR2 as an example. The structure of mGluR2 in an inactive state and a fully active state were extracted from recent Cryo-EM structures (PDB ID: 7EPA [32] and 7E9G [52]). From the inactive state to the fully active state, the most obvious conformational change was the dimerization mode of the two TMDs: the main interaction in inactive mGluR2 was between helix III, and in fully active mGluR2 it was between helix VI [52]. The dimerization mode change implies that the TMDs undergo conformational rotations during the activation process and consequently lead to collisions between TMDs. Therefore, the conformational changes of the two subunits form a coupled relationship instead of occurring synchronously. We obtained the conformational change trajectory and picked 17 conformations (15 intermediates and 2 end points) at equal intervals for each subunit. By combining the conformations of the two subunits, we constructed a two-dimensional conformational change system (Figure 4). Each point in the system represents a possible conformational state during mGluR2 activation. We calculated the folding free energy of each point and then identified the least energy pathway from the inactive state to the fully active state (Figure 4A). The energy landscape of the pathway is shown in the right panel of Figure 4A. The point with the lowest energy may correspond to a stable intermediate conformation. On each side of this point, we found an energy barrier, ΔG_barrier1_ and ΔG_barrier2_ (Figure 4A). The result suggests that mGluR2 activation may be stepwise, consistent with the speculation of previous experiments [53].

Next, we investigated the effect of agonist binding on energy barriers. Based on the two-dimensional conformational change system, we docked one glutamate molecule to the L or R subunits and two molecules to both subunits. The binding free energy was added to the folding free energy of each conformation (Figure 4B–D). We also identified their least energy pathways and described the energy landscapes. When glutamate bound to the L subunit, the two energy barriers increased (Figure 4B). When the agonist bound to the R subunit, only the ΔG_barrier2_ was significantly lowered (Figure 4C). In the case of agonists binding to all subunits, both ΔG_barrier1_ and ΔG_barrier2_ were reduced (Figure 4D). These results reveal the energetic mechanism of asymmetric mGluR2 activation and demonstrate the important role of dual agonist binding.

## 4. Discussion and Conclusions

**Figure 5:**
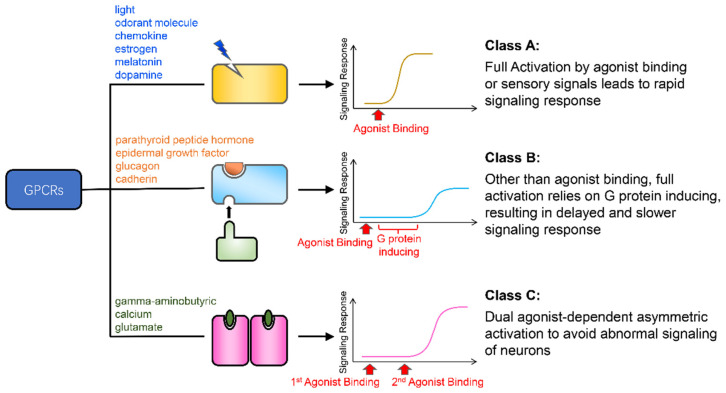
Summary of the conclusions of the work. The yellow, light blue, and magenta rectangles represent the class A, B, and C GPCRs, respectively. The blue flash, orange semicircle, and green oval are the agonists of these three classes of receptor. The text to the left of the cartoons lists examples of signals that activate these receptors. The curves to the right of the cartoons are models of the signaling response curves of these receptors.

The relationship between structure, energy, and function is an important topic in GPCR research [1,2,3,9,13,14]. Here, we described the energy landscapes of the activation process of three GPCRs: *β*2AR, GCGR, and mGluR2. From an energetic perspective, we explained how the kinetic features of the activation free energy profiles of these three GPCRs reveal the deeper nature of their functions (Figure 5). The full activation of *β*2AR by agonist binding leads to a fast response speed and a high signal strength, which fulfills the body’s need for a rapid response to adrenaline. GCGR requires the large ECD to bind the glucagon peptide signal molecule. Other than agonist binding, the full activation of GCGR also relies on the induction of G protein, which results in a delayed and slower signaling response behavior. This is consistent with the requirements for blood glucose regulation. mGluR2 transmits neural signals. Its dual agonist-dependent asymmetric activation mode helps the body to avoid abnormal neuronal signaling. Overall, these results reveal the internal consistency between the structure, function, and energy of the three GPCRs.

The activation models we constructed in this study considered the effect of a single event on the activation of a GPCR. In reality, the full activation of a GPCR is related to several factors, such as the membrane lipid composition, potential G-protein regulators, pH, salt gradients, nucleotide release, etc. The efficiency of signal transmission is not only dependent on the activation of GPCRs but is also related to the downstream factors of the signal pathway. Limited by the structural data and computational tools, such a single-variable model can help us accurately simulate the role played by a single factor in a complex physiological response activity. Our result emphasized the characteristic factors of the activation of these three GPCRs but did not imply that other factors are unimportant. For example, G-protein coupling can further stabilize the fully activated conformation of *β*2AR [54], and glucagon binding can induce the rapid deubiquitylation of GCGRs and promote the recycling of GCGRs [42]. Moreover, although not quite clear, the asymmetric activation of mGluR2 may also be related to the induction of G protein since class C GPCRs have a symmetric dimer interface in the absence of G protein [32,55,56,57]. A more accurate study of the activation mechanism of GPCRs requires more complex models considering the coupling of more factors, which will be a challenge.

These conclusions may also apply to other GPCRs and proteins, but the complexity of biological systems dictates that exceptions always exist. For example, rhodopsin, a representative of class A GPCRs, is an important photoreceptor located in the retina. It adopts an activation mode where the absorption of photons leads to retinal isomerization and provides the energy for the receptor to cross the energy barrier [15]. This mode is different from that of *β*2AR, reducing the energy barrier through agonist binding to the transition state. In addition, the activation of class A GPCRs can be fine-tuned by different ligands or different ligand concentrations. For example, chemokine receptors can be activated rapidly by binding the super-agonist [6P4]CCL5 [58]. Moreover, they can be activated with a lower efficacy by the partial agonist Met-RANTES [59]. In addition, the constitutive activity [60] and the biased signaling [61] of some GPCRs complicate the understanding of the activation processes of all GPCRs. The conclusions we obtained in this work are based on a desirable condition. In order to draw more general conclusions about the mechanism of GPCR activation, we need to take into account these exceptions and perform more experiments.

## Data Availability

Not applicable.
